# Teaching happiness to students – implementation and evaluation of a program aiming at promoting wellbeing in elementary schools

**DOI:** 10.3389/fpsyg.2024.1289876

**Published:** 2024-02-08

**Authors:** Tobias Rahm, Nicole Oberlehberg, Axel Mayer

**Affiliations:** ^1^Institut für Pädagogische Psychologie, Technische Universität Braunschweig, Braunschweig, Germany; ^2^Abteilung für Psychologie, Universität Bielefeld, Bielefeld, Germany

**Keywords:** Positive Education, elementary school, wellbeing, quality of life, program evaluation, happiness lessons

## Abstract

Since the beginning of the COVID-19 pandemic, the prevalence of mental disorders in children and adolescents has increased significantly. Evidence shows that childhood mental disorders can have serious consequences on psychosocial, cognitive, and physical development. Approaches from Positive Education go further than the urgently needed prevention of mental disorders by aiming directly at promoting subjective, psychological, and social wellbeing. The present study describes the implementation of a brief program to promote wellbeing in 15 elementary schools. For this purpose, in a regular university seminar, students of teaching and educational science were instructed to give 11 “happiness lessons” for fourth graders in a team of two and in the presence of the class teacher over the course of 3 months. Quantitative data were collected from children and parents in the treatment group classes and in the parallel classes serving as the waiting control group at four measurement points (pre, post, 1- and 2-month follow-up). We assessed psychological wellbeing, negative emotions and moods, parent support and home life, perception of the school environment, and self-esteem of the children with established instruments with versions for children and their parents and the frequency of positive and negative emotions of the children in self-report only. Additionally, we applied *ad hoc* items on subjective perception of the project and open questions in the treatment group. Data were analyzed with EffectLiteR using multigroup structural equation models. Results showed a small significant effect for negative emotions with the children's data and a medium effect for psychological wellbeing in the perception of the parents at the 1-month follow-up. Interaction effects suggest that lower baseline levels in parent support and home life and self-esteem would increase the treatment effect for these constructs. The need for more grounded framework in positive education and the inclusion of more qualitative methods as well as suggestions to improve the program in the sense of a whole school approach are discussed.

## 1 Introduction

Promoting the mental health and wellbeing of children and adolescents is becoming a stronger focus in society. Current prevalences of mental disorders in childhood and adolescence indicate a clear need for action. For example, the rates in Germany have increased from 9.9% to 17.8% since the beginning of the Corona pandemic (Ravens-Sieberer et al., 2022). It has already been widely shown that mental disorders in childhood can have serious consequences on psychosocial, cognitive, and physical development (e.g., Schulte-Körne, 2022). At the latest with the emergence of positive psychology at the turn of the millennium (Seligman and Csikszentmihalyi, 2000), the focus shifted from the mere absence of psychological limitations to a state of holistic wellbeing as the desirable goal. The Sustainable Development Goals (SDGs) of the United Nations also take this into account. Thus, SDG 3 reads: “Ensure healthy lives and promote wellbeing for all at all ages” (https://www.un.org/sustainabledevelopment/health/). The World Health Organisation (2020, para 1) current definition of health also clearly states that mental health is much more than the absence of mental health problems: “Health is a state of complete physical, mental and social wellbeing and not merely the absence of disease or infirmity.” The active promotion of the wellbeing of children and adolescents is thus of great importance.

High wellbeing is associated with numerous desirable correlates such as sociability and activity, altruism, love of self and others, productivity, a strong immune system, effective conflict solving skills, and resilience (Lyubomirsky et al., 2005; Kansky and Diener, 2017). Furthermore, individuals with high levels of wellbeing experience fewer psychological problems and are more effective at coping with negative experiences (Kansky and Diener, 2017). In their model about the origins of happiness, Clark et al. (2018) describe determinants of adult life satisfaction. Driven by empirical evidence the authors show that experiences and circumstances in family and schooling affect intellectual, behavioral, and emotional outcomes for the children which in turn influence adult outcomes (such as income, crime, or health) as well as adult life satisfaction. Just as some aspects like high income or good parental health have a positive influence, other factors like low income or parental conflicts have a corresponding negative influence. Many of those factors are subject of complex political decisions, others can be targeted by educational processes.

For universal promotion of children's wellbeing, schools provide the optimal context because they are one of the most important developmental sites in childhood and adolescence (Eccles and Roeser, 2011) and large numbers of children can be reached by programs (Seligman et al., 2009). In addition, this is where society has the greatest impact on the upcoming generations.

Positive Education, a subfield of Positive Psychology, seeks to actively and passively promote the wellbeing of school children in addition to the traditional academic goals (Seligman et al., 2009). Positive Education programs address many different content areas, such as mindfulness, resilience, character strengths, optimism, self-worth, emotion regulation, flow experience, goal achievement, and more, depending on their scope and duration (see Bott et al., 2017). The underlying constructs of wellbeing differ from study to study and include Diener's (2000) subjective wellbeing, Ryff's (1989) psychological wellbeing, various conceptualizations of flourishing (cf. Hone et al., 2014), and health-related quality of life (Ravens-Sieberer et al., 2014).

Preparatory work for this article was conducted as part of the unpublished master's thesis by Oberlehberg (2023) and the associated pre-registration with OSF (https://doi.org/10.17605/OSF.IO/K3TWR). Only the children's quantitative data and only from the first three measurement points were analyzed in the thesis.

### 1.1 Previous findings on Positive Education programs

In a meta-analysis of nine studies, Tejada-Gallardo et al. (2020) found short-term significant improvements in depressive symptoms (*g* = 0.28), subjective wellbeing (*g* = 0.24), and psychological wellbeing (*g* = 0.25) with multicomponent positive psychological interventions with 6 to 18 sessions that were delivered within 6 to 30 weeks to children aged 10 to 18 years in different countries. Long-term effects (about 6-month follow-up) remained significant for psychological wellbeing (*g* = 0.44) and depressive symptomatology (*g* = 0.31), but not for subjective wellbeing.

Another meta-analysis by Taylor et al. (2017) focused on social and emotional learning interventions and examined 82 studies. The sample included children from kindergarten to high school (*N* = 97,406) from different countries. Results showed that children in the intervention group had better long-term scores (at least 6-month follow-up) in social-emotional skills (*g* = 0.23), attitudes (*g* = 0.13), and indicators of wellbeing (*g* = 0.13 to 0.16) compared to a control group, regardless of the children's ethnicity, socioeconomic background, or school location.

Adler's (2016) dissertation work implemented a 15-month positive-psychology curriculum in schools in three different countries with nested randomized control trial designs. The curriculum targeted ten non-academic “life skills” namely mindfulness, empathy, self-awareness, coping with emotions, communication, interpersonal relationships, creative thinking, critical thinking, decision making and problem solving. Teachers were trained intensively to not only convey these skills directly but also to infuse their academic subjects with the ten life skills. Effects of the curriculum were tested in comparison to a placebo curriculum with a content of basic nutrition, psychology, and human anatomy. In the first study, the programs were implemented in 18 secondary schools in Bhutan with *N* = 8,385 participating school children aged 10 to 24 years (*M* = 15.1, *SD* = 2.2). As a measure for children's wellbeing the EPOCH questionnaire (Kern et al., 2016) was used. Here, the positive psychology curriculum significantly improved children's wellbeing (*d* = 0.59) and school performance (*d* = 0.53) from pre to post. These effects were evident 12 months after the end of the program. In the second study, he examined 70 upper secondary schools in Mexico with *N* = 68,762 school children aged 13 to 26 years (*M* = 16.2, *SD* = 1.1) in the same way. This also showed a significant effect of the positive psychology curriculum on children's wellbeing (*d* = 0.41) and academic achievement (*d* = 0.36). In Study 3, he implemented the curricula in 694 secondary schools in Peru with *N* = 694,153 school children aged 11 to 28 years (*M* = 15.4, *SD* = 0.8). Again, there were significant effects on children's wellbeing (*d* = 0.24) and academic achievement (*d* = 0.19). In all three studies, persistence, engagement, and quality of interpersonal relationships also emerged as the strongest wellbeing-related predictors of academic achievement.

In 2006, Seligman et al. (2009) implemented a Positive Education curriculum at Geelong Grammar School in Australia (Bott et al., 2017). Positive psychology interventions and psychoeducation based on the PERMA model (Seligman, 2011) were not only introduced as a separate subject twice a week, but were also implicitly included in the regular curriculum. In addition, all staff and parents were integrated into the project (for more information, see Frydenberg et al., 2017). In the evaluation, school children in grades 9 to 11 showed significantly fewer depressive and anxiety symptoms, as well as significantly higher scores in wellbeing, growth mindset, sense of purpose, and hope, compared to a matched control group (Bott et al., 2017). In this context, Dubroja et al. (2016) examined the effectiveness of parent involvement in an initial pilot study. For this, *n* = 22 parents participated in a three-day course with positive psychological interventions, and *n* = 11 parents formed the waiting control group. Parents reported significantly higher wellbeing at post (η*p*^2^ = 0.32) and 2-month follow-up measurement time points (η*p*^2^ = 0.31). In addition, the perceived parent-school relationship improved significantly from the parents' perspective at the post measurement time point (η*p*^2^ = 0.39). No significant improvements were shown in the perceived parent-school relationship at the follow-up measurement time point, in parent ratings of child wellbeing, and in the perceived warmth of the parent-child relationship. Previous research has thus shown positive effects of Positive Education in different countries on various areas of wellbeing as well as on academic performance with small to medium effect sizes.

In Germany, many programs exist that are dedicated to preventing individual undesirable behaviors such as bullying or exposure to violence or mental illness. Many of these programs include elements of self-empowerment and are thus also likely to promote the overall wellbeing of participants. However, explicit Positive Education programs are few in number and have been evaluated only to a limited extent. The best known in Germany is “Schulfach Glück” for which there is some evidence of improvement in sense of coherence, sense of consistency, self-esteem, and subjective wellbeing from individual studies (Fritz-Schubert, 2017). Another program from the German-speaking area that can be assigned to Positive Education is the “Curriculum Schulfach Glückskompetenz” by Mathes (2016a,b), which is available in the form of two published books. It is already being used in a variety of formats, but has not yet been systematically evaluated.

### 1.2 Aims of the present study

Various goals were pursued with the implementation of the pilot project “Glückskompetenz in der Grundschule (GlüGS-Projekt)” (Happiness Competence in Elementary School). First, a university course was developed in which students could be qualified to carry out a positive education program to promote wellbeing in school. The overall goal was to promote the wellbeing of children participating in the intervention. In support of the content of the 11 “happiness lessons,” measures were taken to involve parents^1^ in order to increase the chance of effectiveness. Furthermore, the class teachers of the participating classes were encouraged to implement additional wellbeing-promoting content, attitudes, behaviors, and rituals during the intervention as well as after its end. The present study aimed at evaluating the impact of the intervention on the participating school children. The original program was developed for third and fourth grade. We chose to include only fourth classes as we expected higher data quality from the questionnaires due to a better understanding of the items and more reflective competencies.

### 1.3 Hypotheses

We postulate that the program described below has positive effects on the following constructs for participating fourth graders compared to non-participating fourth graders:

- *psychological wellbeing* (H1)
- *negative emotions and moods* (H2)
- *parents support and home life* (H3)
- *perception of the school environment* (H4)
- *self-esteem* (H5)
- *frequency of positive emotions* (H6)
- *frequency of negative emotions* (H7)

We assume that the positive effects occur immediately after the treatment (post) and at one-month and two-month follow-up (1mfu and 2mfu). For the Hypotheses H1 to H5 we also postulate positive effects in the parents' assessment of their children (H1_parents_ - H5_parents_).

Additionally, we assume a positive perception of the program measured quantitatively with *ad hoc* satisfaction items and qualitatively with open questions for participating children, parents, and teachers.

## 2 Materials and methods

### 2.1 Sample

The project was introduced in a mandatory online meeting of the department of school affairs with about 40 participating elementary school principals. Over the next few weeks, 16 schools signed up to participate. Each school participated with two fourth-grade classes, one of which was assigned to the treatment group and the other to the waiting control group. Two of the participating schools had only one fourth-grade class; of these, one was assigned to the treatment group and one to the control group. For organizational reasons, the assignment was done by the schools themselves and could not be randomized. A total of *N* = 491 school children in fourth grade and *N* = 245 parents participated. Table 1 shows the distribution of the sample at baseline measurement. As can be seen, there are more female than male fourth graders and the control group has more German speakers than the treatment group. The average age for schoolchildren in fourth class in Germany is 9 years. Most of the answering parents were mothers. The teachers of the classes were asked to provide some basic information about themselves and their classes. From the 30 teachers, 3 did not participate. From the remaining 27 only one teacher was male. Their years of experience in teaching differed from 2 years to 35 years (*M* = 20.88, *SD* = 8.67). The classes had between 14 and 24 schoolchildren (*M* = 18.59, *SD* = 2.75). The intervention was carried out by 25 trained university students (7 male and 18 female) from 1st to 7th semester studying teaching or educational sciences. For ten classes, a pair of students could be realized, while in five classes only one student delivered the intervention. When assigning students to schools, care was taken to ensure that at least one student with increased experience was appointed.

**Table 1:** Sample description (at baseline).

**Children**		**Treatment (*n =* 249)**		**Control (*n =* 242)**	
**Demographic variable**	**Category**	**Frequency**	**Percentage**	**Frequency**	**Percentage**
Gender	Male	114	45.8%	122	50.4%
	Female	135	54.2%	120	49.6%
German as native language	Yes	188	75.5%	197	81.4%
	No	61	24.5%	45	18.6%
Mostly German at home	Yes	199	79.9%	207	85.5%
	No	50	20.1%	35	14.5%
**Parents**		**Treatment (*****n** =* **136)**		**Control (*****n** =* **109)**	
**Demographic variable**	**Category**	**Frequency**	**Percentage**	**Frequency**	**Percentage**
Parental role	Mother	110	80.9%	90	82.6%
	Father	18	13.2%	14	12.8%
	Other^*^	8	5.9%	5	4.6%
German as native language (child)	Yes	121	89.0%	102	93.6%
	No	15	11.0%	7	6.4%
Mostly German at home	Yes	126	92.6%	105	96.3%
	No	10	7.4%	4	3.7%

^*^The category “Other” includes 4 respondents who did not assign themselves and 9 cases in which different genders were indicated at different measurement points.

### 2.2 Structure and content of the seminar for the university students

Students of teaching and educational science at a German university were able to register voluntarily for the project as part of an elective module. In an introductory event with the participation of students, schools, school authorities, the faculty of the university as well as the regional newspaper, the project with its contents and processes was explained and contextualized within the larger framework of the Positive Education movement. In preparation for the weekly seminar, students viewed the learning video and read the instructional planning for the upcoming lesson. In the seminar, the past lesson was first reflected on in a joint discussion, with each student briefly reporting on the process, highlights, and difficult situations. Subsequently, the upcoming lesson was reviewed on the basis of the lesson plan. Didactic advice was given, potentially difficult situations and questions from the students were addressed, and some of the exercises were tried out. In addition, the prepared teaching materials were handed out. The seminar was allocated in the module “Education for Sustainable Development,” in which students can earn 2 credits (according to the European Credit Transfer and Accumulation System - ECTS) after attending the 12 weekly sessions, conducting the 11 happiness lessons, filling out a short reflection sheet after each lesson and writing a short final reflection.

### 2.3 Structure and content of the program

The original program “Curriculum Schulfach Glückskompetenz” (Mathes, 2016a,b) is available in two books. Part I explains the building blocks with psychological and pedagogical backgrounds and describes individual learning units in a way that can be implemented by teachers after only a short adaptation to their own context. Part II contains the required materials such as stories, letters to parents, worksheets, etc., many of which can be used directly as copy templates. Mathes (2016b) pursues several goals with her program. These include helping schoolchildren grow into resilient, emotionally stable, and authentic individuals through taking responsibility for their wellbeing, developing social and emotional skills, and becoming competent in dealing with future difficult situations as well as stress and anxiety. The program was developed for schoolchildren in third and fourth grade. For the present study, the published concept was condensed into 11 specified 45-min sessions. Since bachelor students should be able to carry out the program, these sessions were elaborated in the form of tables where every section of the lesson was explained in detail (with an indication of duration, exemplary word-for-word instructions, required materials, and didactic advice). For each learning unit, an ~20-min learning video was produced for the university students.

All learning units had the same structure. For the opening welcome, each child jumped onto one of three symbol pictures, calling out his or her name, and was greeted by the university student with a fist bump, a heart formed with the fingers in front of the chest, or a little dance depending on the selection of the symbol picture, and the words “Hello [first name], glad you're here!” The introduction to the topic of the lesson was mostly done through short stories or picture cards followed by a class discussion. In addition, various active exercises were carried out to anchor the learning content (movements, handicrafts, painting, etc.). Finally, a short summary was given by the university students and a letter was handed out for the parents, in which the contents of the lesson were summarized on one page and suggestions for applications in the family were given. At the end, the concluding ritual was performed together in the form of a chant with simple movements. The contents of the 11 lessons can be found with sample exercises and tips from the letters to parents in Table 2.

**Table 2:** Contents of the program.

**Lesson title**	**Content and competencies**	**Sample exercise**	**Tip in parent letter**
1. What is happiness?	Introduction to the topic of wellbeing, working out individual definitions and factors of happiness. Homework: keep a Happiness Diary for 1 week.	Children work in groups of four, each child writes his or her own definition of happiness on a quarter of a placemat, followed by sharing ideas and collecting three key statements for each group	Discuss definitions of happiness in family.
2. Recognizing and increasing happiness	Becoming aware of individual attention focus on positive and negative events; collecting positive memories which are hung up in the classroom.	A story introduces the Yellow Backpack, where protagonists collect their good memories. A poster is created where this is done with the class.	Make a family treasure chest of positive memories (e.g., memories or objects written down).
3. Happiness is learnable and trainable	Definition of neuroplasticity and realization that wellbeing can be trained; reflection on individual frequency of different emotions. Homework: draw a personal Garden of Emotions.	Children make a Garden of Emotions. Each child writes a positive or negative feeling on a flower. On the back, they write down a situation that matches the feeling. All the flowers are put into a made-up garden.	Strengthen focus on positive events.
4. Relaxation and mindfulness as a prerequisite for positive perception	Experience inner peace and mindful perception; allow, accept and objectively evaluate feelings in a relaxed state; establish dream journey and imaginary place of wellbeing; reflect on the exercise and consolidate the place of wellbeing.	The imagined place of wellbeing is painted by each child, and the feelings associated with it are to be memorized. The picture can be used for emotion regulation and relaxation.	Perform a dream journey.
5. Empathy and emotional contagion	Theoretical and practical learning that the mood of others influences one's own mood; psychoeducation on the topic of empathy.	Good Mood Alley: Children stand in two rows facing each other and put their hands together in the middle to form a roof. One child walks from the back to the front through the alley and is smiled at by everyone.	Reflect on own beneficial friendships.
6. Altruism and doing good	Recognize the connection between altruism and wellbeing; reflect on own altruistic acts. Homework: Do something good for someone else.	Watch videos or pictures on helpfulness and reflect on your own helpfulness and the feelings associated with it.	Reflect with family on own and experienced altruism.
7. Appreciation and social competence	Learning to give and receive compliments. Homework: Write 10 anonymous compliment cards for classmates.	Each child receives a Warm Shower Card and writes a compliment to a classmate. Compliments are read out in a large group. Afterwards reflection on the exercise and feelings.	Reflection on self-appreciation and appreciation by others.
8. Gratitude	Each child receives a personal gratitude card from the teacher; individual reasons for gratitude are collected	Chain of gratitude: The children write on strips of paper what they are grateful for. These are glued together and hung up as a chain in the classroom.	Conscious gratitude in the family, e.g., through a thank-you pinboard.
9. Perceiving emotions in the body	Recognize and name emotions in pictures; recognize the connection between body sensations/ facial expressions/ posture and emotions; learn to influence own emotions.	Children choose an emotion card and name the feeling and how they can recognize the feeling.	Experience the connection between body posture/cognitions and emotions.
10. Self-confidence and self-efficacy	Learn that social comparisons can be dysfunctional; reflect on own strengths, positive qualities and goals.	Fill in the strengths tree: What am I good at? What are my strengths? Who gives me strength? Where and with whom do I feel safe and secure? What are my goals? a.o.	Reflect on own strengths, positive qualities, wishes and goals.
11. Final lesson	Reflection on past lessons, focusing on positive experiences; developing a habit to sustainably increase wellbeing; conclusion.	Children reflect on the happiness lessons and tell what they particularly liked or liked best. Results are written down.	Develop detailed planning of own and family happiness habits.

### 2.4 Measures

For the evaluation of the present program, we decided to focus on the health-related quality of life, since an internationally normed and established measurement instrument for use in elementary schools with self- and external assessment scales exists for this construct (KIDSCREEN; Ravens-Sieberer et al., 2014). The same is true for self-esteem (KINDL-R; Ravens-Sieberer and Bullinger, 1998a,b). Additionally, we assessed the frequency of positive and negative emotions (SPANE; Rahm et al., 2017, adapted for children). Finally, participating fourth graders, their parents and their class teachers answered self-constructed items on the subjective treatment perception as well as open questions. The previously found psychometric properties of the scales are reported together with the ones found in this study in the results section. The reliabilities of all subscales were found to be satisfactory or better (Cronbach's α > 0.70).

#### 2.4.1 Health-related quality of life

To assess aspects of the wellbeing of the children we used the KIDSCREEN-52 (Ravens-Sieberer et al., 2014) that measures the health-related quality of life on originally ten dimensions. In this study only four dimensions were used: The subscale *psychological wellbeing* records life satisfaction (example item: “Has your life been enjoyable?”) and positive emotions (“Have you been in a good mood?”). The *moods and emotions* subscale asks about negative moods and feelings (“Have you felt that you do everything badly?”). The subscale *parent support and home life* measures the relationship with parents, the atmosphere in the family, and the quality of interaction [“Have your parent(s) understood you?” or “Have you been happy at home?”]. The subscale on *school environment* deals with learning, concentration, attitude toward school, satisfaction with school performance, and the child's relationship with the teacher (“Have you been happy at school?” or “Have you been able to pay attention?”). In each case, the questions refer to the past week. Each subscale has six to seven items, each of which is answered on a 5-point response scale from “not at all” (1) to “extremely” (5) or “never” (1) to “always” (5). The items of the *moods and emotions* subscale are all negatively worded. The values of the items were recoded so that higher values indicate higher wellbeing here as well. In addition, one item from the *physical health* subscale was used (“In general, how would you say your health is?”), answered on a 5-point response scale from “poor” (1) to “excellent” (5). For the KIDSCREEN, in addition to the self-assessment version, there is also one for external assessment by parents; the corresponding scales were also used in this study.

#### 2.4.2 Self-esteem

In order to assess the children's *self-esteem*, the KINDL-R subscale of the same name was used (Ravens-Sieberer and Bullinger, 1998a,b). The subscale consists of four items, each referring to the last week (example item: “In the last week I was proud of myself”) and can be answered with a 5-point response scale from “never” (1) to “always” (5). In addition to the self-assessment version, the KINDL-R is also available for external assessment by parents; the corresponding scale was also used in this study.

#### 2.4.3 Frequency of positive and negative emotions

The *frequency of positive and negative emotions* is assessed with an adopted version of the Scale of Positive And Negative Experiences (SPANE; Original: Diener et al., 2010; German version: Rahm et al., 2017). The instrument distinguishes between two subscales, one for positive and one for negative experiences. We adapted the scale for the purpose of this study to make it more comprehensible for fourth grade children using four more general feelings (e.g., “pleasant” or “unpleasant”) and six more specific feelings (e.g., “joyful” or “sad”) evenly distributed to both subscales with five items each. Participants were asked how often they experienced the given emotion in the past week and to answer on a 5-point scale from very rarely (1) or never to very often or always (5).

#### 2.4.4 Subjective perception of the happiness lessons for the treatment group at t2

For the assessment of the *subjective perception of the happiness lessons* we delivered seven (for children) or eight (for parents and teachers) *ad hoc* items about potentially perceived effects of the participation in the program only to the treatment group and only at post measurement point (e.g., “I learned important things in the happiness lessons.”). The items were to be rated on a five-point scale from not true (1) to true (5). We also used 3 open questions to be answered qualitatively. All item wordings can be found in the results section.

### 2.5 Procedure

**Figure 1:**
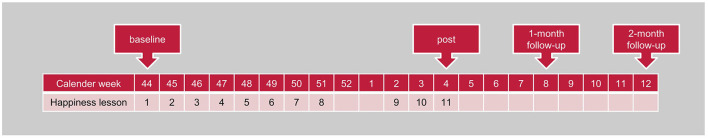
Program sequence and measurement points.

The program was delivered by the university students every school week starting directly after the autumn break in October 2022 until midterm at the end of January 2023 in the treatment group. The questionnaires for the children were answered unanonymously in the classroom at the beginning of the first lesson (baseline), at the end of the last lesson (post) and one resp. two month after the last lesson (1mfu and 2mfu). The participants of the control group answered the questionnaire on the same day as the treatment group. Parents received an e-mail with a link to an online questionnaire that could be answered for 1 week, starting on the day after the children were assessed. Due to data security, we did not collect e-mail-addresses but asked the class teachers to forward an e-mail to the parents and a reminder about 3 days later. Figure 1 shows the sequence of measurement points and lessons of the program.

All parents of the participating fourth graders were informed in detail about the voluntary nature of the participation, the measures taken to maintain data security, and the purpose of the study. They gave their written informed consent for further data processing. The study was approved by the Ethics Committee of the Faculty of Life Sciences, Technische Universität Braunschweig (FV-2022–19) and the Department of School Affairs.

### 2.6 Data analysis

For the descriptive analysis of the data, the scale scores were calculated according to the instructions of the respective authors. For the KIDSCREEN, the syntax for SPSS provided on the website was used, resulting in Rasch-scaled *T-*values with a mean of 50 and a standard deviation of 10 referring to the European norm sample. For the KINDL-R, the scale score was formed according to official syntax and then transformed to a 100-scale. For the SPANE, mean values were computed. For the KIDSCREEN, appropriate norm values are available, with which the values from the baseline measurement were compared. The evaluation of the *ad hoc* items for subjective assessment was done on an item basis. The data preparation and descriptive statistics were performed with SPSS 25. For the open answers to the questions about the subjective perception of the program, the responses were categorized by content.

Due to different sample sizes, the analytical procedure differed for parents' and children's data. Therefore, the analytical procedures for children and parents are reported separately in the following.

#### 2.6.1 Children data

**Figure 2:**
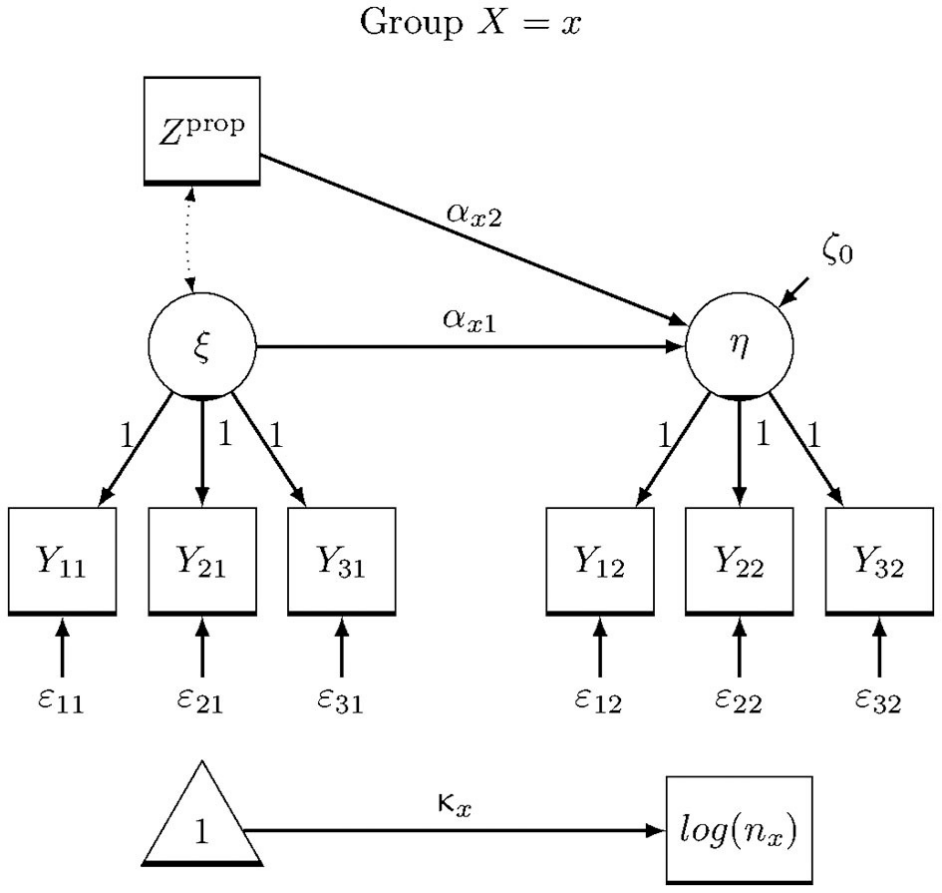
Group-specific path diagram of the multigroup structural equation model that is the basis for the computation of average and conditional effects using EffectLiteR. *Y*_it_ stands for item parcel i at time point t of the corresponding scale (e.g., *psychological wellbeing*), ε_it_ are measurement error variables, η is the latent dependent variable at posttest (or follow-up respectively), ξ is the latent pretest, and Z^prop^ is the logit transformed propensity score. The model includes parameters for the stochastic group sizes κ_x_ that are used in the computation of the average effect. In both groups, the regression of η on ξ and Z^prop^ is estimated, where α_x1_ and α_x2_ denote the group-specific regression coefficients and ζ_0_ is the residual.

The data of the children were assessed by means of questionnaires in the classroom, which resulted in a high level of participation. Some children were absent at individual measurement times for example due to illness. For the main analysis of the children's data, therefore, multi-group structural equation models with latent variables including a propensity score, and the formation of item parcels could be applied. Figure 2 shows the exemplary path diagram of the multigroup structural equation model. The seven constructs *psychological wellbeing, negative emotions and moods, parent support and home life, perception of the school environment, self-esteem*, and the *frequency of positive and negative emotions* are the latent dependent variables. For each measurement time point (post, 1mfu, 2mfu) one model was built for every dependent variable – 21 models in total. As independent variables, the respective latent baseline measurement of the corresponding dependent variable, the dichotomous treatment variable, the interaction between baseline and treatment, and a propensity score were included in the models. Analyses were computed using EffectLiteR (Mayer et al., 2016, 2020) in R Core Team (2022).

The items of the *self-esteem* scale of the KINDL-R were used to define the corresponding dependent latent variables at post, 1mfu and 2mfu and the corresponding independent latent variable at baseline measurement. Accordingly, the items of the two subscales of the SPANE were used to define the dependent latent variables *positive emotions* and *negative emotions* at post, 1mfu and 2mfu and as the corresponding independent latent variables at baseline measurement. For the Rasch-scaled items of the four KIDSCREEN scales used, item parcels were formed. The procedure offers psychometric advantages by taking measurement errors into account as well as advantages for model estimation (Little et al., 2022). Parcels were created from two items each (or three items once for the *moods and emotions* subscale) prior to analysis. They were formed according to the recommendation of Little et al. (2013), using the balancing approach. The required loadings for the balancing approach were calculated using the package lavaan (Rosseel, 2012) with a confirmatory factor analysis.

Baseline measurements were z-standardized for a better interpretability of interaction effects. For missing values, the full information maximum likelihood (FIML) method was used. For the measurement models, either tau-equivalent or tau-congeneric models were used depending on the model fit. Glass's delta was calculated as effect size, the type I error rate was set at 0.05 for all analyses, and all *p*-values are two-sided.

To control for selection bias, a propensity score was formed (Schafer and Kang, 2008). This is often used in non-randomized studies and denotes the individual treatment probability depending on relevant covariates (Rosenbaum and Rubin, 1983). In the present work, covariates for the propensity score were selected through theoretical and statistical considerations. Variables were included in the propensity score for statistical reasons if the standardized mean difference (SMD) of baseline measurements between groups was >0.1 (see Table 3 for descriptive statistics and SMD of covariates). For each of the 21 models, a separate propensity score was calculated. Physical health and gender were used as covariates based on empirical associations with child wellbeing (cf. Erhart et al., 2009). In addition, the scale scores of each of the other six scales were selected as covariates for the respective propensity score based on theoretical and empirical associations with child wellbeing and statistical group differences from baseline. Additionally, the two items on native language and language in the parental household were included, as a poorer understanding of the German language could possibly have an influence on the response. The respective propensity score was logit transformed and included as a covariate in the analysis (cf. Mayer, 2019).

**Table 3:** Standardized mean differences of the covariates of the propensity score (children).

	**Control**		**Treatment**		
	***n***	***M (SD)***	***n***	***M (SD)***	***SMD***
Physical health	224	4.04 (0.86)	224	4.12 (0.79)	0.10
Psychological wellbeing	220	54.45 (8.05)	220	54.38 (8.02)	0.01
Moods and emotions	217	48.63 (9.68)	212	49.46 (8.51)	0.09
Parent support and home life	203	58.99 (9.90)	209	57.52 (9.73)	0.15
School environment	211	57.07 (10.35)	219	56.17 (9.13)	0.09
Self-esteem	222	73.73 (19.47)	219	70.66 (19.50)	0.16
Positive emotions	223	3.96 (0.72)	219	3.92 (0.72)	0.06
Negative emotions	221	1.89 (0.70)	218	2.05 (0.81)	0.21
	***n***	**%**	***n***	**%**	
**Gender**					
Female	123	50.4	116	45.8	
Male	121	49.6	137	54.2	
**Native language**					
Not German	45	18.6	61	24.5	
German	197	81.4	188	75.5	
**Language at home**					
Not German	35	14.5	50	20.1	
German	207	85.5	199	79.9	

SMD, standardized mean differences; M, mean; SD, standard deviation.

An a priori power analysis based on Monte Carlo simulations using the packages MASS (Venables and Ripley, 2002), EffectLiteR (Mayer et al., 2016), and SimDesign (Chalmers and Adkins, 2020) revealed that an effect as small as *d* = 0.2 can be revealed with a sample size of approximately *N* = 240 per group with an estimated power of 0.80.

In addition, the prerequisites for structural equation models were tested. To test whether the observations were independent, the intraclass correlation coefficients (ICC) of the dependent variable were calculated. These were below ICC = 0.1 for the self-esteem, SPANE-P, and SPANE-N scales, and for all but one of the item parcels of the KIDSCREEN scales, indicating that the class of the children accounted for only a negligible portion of the variance. Independence of the observations could thus be assumed, and no cluster variable was used in further analysis to account for the multilevel design. Because a multivariate normal distribution could not be assumed for any of the dependent variables according to the test of Mardia (1970), robust Huber-White standard errors were chosen for the analyses. Depending on whether variance homogeneity could be assumed, either homogeneous or heterogeneous variances were specified for the analyses.

#### 2.6.2 Parents data

Parents were asked to participate in online questionnaires by e-mail via the class teachers. Due to this procedure, the response rate was considerably lower than for the children's data collection. In addition, data from all four measurement time points are only available in comparatively few cases; in many cases, responses were only received at one, two, or three measurement time points. In some cases, individual parents appear to have taken turns answering the survey despite being instructed otherwise. Because of the smaller sample size, the procedure for the main analysis was simplified compared to the procedure with the children's data. Instead of latent variables, the manifest variables (i.e., the scale values formed as described above) were used throughout. The dependent variables were the five external assessment scales *psychological wellbeing, moods and emotions, parent support and home life, school environment*, and *self-esteem*. For each measurement time point (post, 1mfu, 2mfu), one model was built for every dependent variable - a total of 15 models. As independent variables, the respective baseline measurement of the corresponding dependent variable, the propensity score, the dichotomous treatment variable, and the interaction between baseline and treatment were included in the models. The propensity score was formed as described at the children data section – Table 4 shows the descriptive statistics and SMD of covariates for the parents' data. Item parcels were not created due to the smaller sample size. Analyses were computed using EffectLiteR based on an ordinary least squares estimator instead of the multigroup structural equation modeling approach used for the children's data with latent variables. Baseline measurements were z-standardized for a better interpretability of interaction effects. Missing values were excluded listwise. Glass's delta was calculated as effect size, the type I error rate was set at 0.05 for all analyses, and all *p*-values are two-sided.

**Table 4:** Standardized mean differences of the covariates of the propensity score (parents).

	**Control**		**Treatment**		
	***n***	***M (SD)***	***n***	***M (SD)***	***SMD***
Physical health	91	2.05 (0.74)	102	2.23 (0.77)	0.23
Psychological wellbeing	91	51.55 (8.64)	102	50.25 (7.94)	0.16
Moods and emotions	91	47.15 (9.63)	101	46.39 (10.07)	0.08
Parent support and home life	89	51.51 (8.70)	102	48.29 (7.58)	0.40
School environment	91	53.18 (10.08)	102	52.25 (9.19)	0.10
Self-esteem	91	74.18 (18.26)	102	71.57 (13.21)	0.16

SMD, standardized mean differences; M, mean; SD, standard deviation.

## 3 Results

Table 5 shows the scale characteristics for the baseline measurement for children and parents as well as the German norm data of the KIDSCREEN for the age group of 8–11 years (Child Public Health, 2020). While the means for the *psychological wellbeing* and *school environment* scales for children are very close to those of the norm sample, the deviations for *moods and emotions* (here 49.04; norm 53.92) and *parent support and home life* (here 58.25; norm 52.56) are slightly more than half a standard deviation. Regarding the comparison of the parent data with the norm data, it is noticeable that the values in the norm sample are slightly higher for all scales. For the scales *parent support and home life* and *school environment* the differences are smaller, whereas for the scales *psychological wellbeing* (here 50.86; norm 53.15) and *moods and emotions* (here 46.74; norm 49.93) the differences are higher. Suitable comparison data are not available for the other measurement instruments. Internal consistencies are reported as Cronbach's α. As can be seen, for the children's data all values are lower than the ones in the norm samples but are still at least in the acceptable range (>0.70). For the parents' data, Cronbach's α's are closer to the norm values and partly better in the present study.

**Table 5:** Scale characteristics (pre) and norm data.

**Children**										
			**This study**				**Norm sample**			
**Construct**	**Scale**	**# Items**	***N***	***M***	***SD***	α	***N***	***M***	***SD***	α
Psychological wellbeing	KIDSCREEN-PW	6	440	54.41	8.02	0.79	596	56.15	8.05	0.89
Moods and emotions	KIDSCREEN-ME	7	429	49.04	9.12	0.84	598	53.92	10.02	0.86
Parent support and home life	KIDSCREEN-PH	6	412	58.25	9.83	0.77	594	52.56	8.34	0.89
School environment	KIDSCREEN-SE	6	430	56.61	9.75	0.83	594	55.30	10.23	0.87
Self-esteem	KINDL-R-SE	4	441	72.21	19.53	0.71	n.a.			0.70
Positive emotions	SPANE-P	5	442	3.94	0.72	0.81				n.a.
Negative emotions	SPANE-N	5	439	1.97	0.76	0.75				n.a.
**Parents**										
			**This study**				**Norm sample**			
**Construct**	**Scale**	**# Items**	***N***	***M***	***SD***	α	***N***	***M***	***SD***	α
Psychological wellbeing	KIDSCREEN-PW	6	193	50.86	8.28	0.84	603	53.15	8.05	0.90
Moods and emotions	KIDSCREEN-ME	7	191	46.74	9.85	0.86	600	49.93	9.74	0.84
Parent support and home life	KIDSCREEN-PH	6	191	49.79	8.25	0.88	593	50.69	7.91	0.87
School environment	KIDSCREEN-SE	6	193	52.69	9.61	0.86	599	53.87	9.52	0.88
Self-esteem	KINDL-R-SE	4	193	72.80	15.81	0.74	n.a.			0.68

M, mean; SD, standard deviation; α, Cronbach's α.

In the following, we first report the results for the children's questionnaire data and then those for the online questionnaires of the participating parents in regard to the hypotheses. After that we report the results of the *ad hoc* items on subjective assessments for children, parents, and teachers and finally the categorized answers of children, parents and teachers to the open questions.

### 3.1 Children data results

The research hypotheses were tested with 21 structural equation models for the seven scales at three measurement time points. Table 6 shows the adjusted means and standard errors for the scales used per group and measurement time point, as well as the average effects of the treatment and the interaction between the baseline measurement and the treatment condition.

**Table 6:** Effects of the program – children.

**Post**												
**Scale**	**Treatment**			**Control**			**Average effect**			**Baseline x Treatment**		
	***N***	***M***	***SE***	***N***	***M***	***SE***	**Estimate**	***p***	**Glass d**	**Estimate**	**SE**	***p***
Psychological wellbeing	244	4.38	0.04	237	4.29	0.05	0.09	0.090	0.15	−0.01	0.10	0.925
Moods and emotions	244	4.17	0.05	237	4.19	0.06	−0.02	0.761	−0.03	−0.10	0.12	0.398
Parent support and home life	243	4.47	0.04	234	4.45	0.05	0.02	0.657	0.039	**−0.27**	**0.09**	**0.015**
School environment	243	4.16	0.05	234	4.09	0.05	0.06	0.291	0.09	−0.14	0.12	0.228
Self-esteem	243	4.04	0.05	237	3.91	0.06	0.13	0.056	0.17	−0.15	0.10	0.148
Positive emotions	243	4.23	0.05	237	4.15	0.06	0.07	0.342	0.10	0.03	0.12	0.772
Negative emotions	243	1.93	0.06	236	2.06	0.07	−0.13	0.095	−0.17	−0.19	0.13	0.139
**1-month follow-up**												
**Scale**	**Treatment**			**Control**			**Average effect**			**Baseline x Treatment**		
	***N***	***M***	***SE***	***N***	***M***	***SE***	**Estimate**	***p***	**Glass d**	**Estimate**	**SE**	***p***
Psychological wellbeing	243	4.33	0.03	237	4.29	0.05	0.04	0.445	0.07	−0.06	0.11	0.551
Moods and emotions	243	4.20	0.06	237	4.22	0.06	−0.03	0.733	−0.04	0.11	0.14	0.414
Parent support and home life	242	4.47	0.04	235	4.44	0.04	0.03	0.534	0.06	0.02	0.10	0.822
School environment	242	4.16	0.04	235	4.14	0.05	0.02	0.775	0.02	−0.12	0.09	0.208
Self-esteem	244	4.04	0.05	237	3.92	0.06	0.12	0.086	0.16	−0.10	0.10	0.359
Positive emotions	244	4.30	0.05	237	4.22	0.06	0.09	0.195	0.13	−0.07	0.13	0.591
Negative emotions	244	1.85	0.06	237	2.03	0.07	**−0.18**	**0.020**	**−0.24**	−0.07	0.14	0.639
**2-month follow-up**												
**Scale**	**Treatment**			**Control**			**Average effect**			**Baseline x Treatment**		
	***N***	***M***	***SE***	***N***	***M***	***SE***	**Estimate**	***p***	**Glass d**	**Estimate**	**SE**	***p***
Psychological wellbeing	243	4.36	0.04	237	4.37	0.05	−0.02	0.779	−0.03	−0.13	0.13	0.317
Moods and emotions	242	4.20	0.05	237	4.31	0.05	−0.11	0.096	−0.15	0.03	0.13	0.796
Parent support and home life	242	4.48	0.04	235	4.49	0.05	−0.01	0.876	−0.02	−0.10	0.11	0.352
School environment	241	4.07	0.06	235	4.10	0.06	−0.03	0.619	−0.04	0.09	0.11	0.413
Self-esteem	240	4.07	0.06	236	4.08	0.06	−0.02	0.841	−0.02	**−0.29**	**0.14**	**0.041**
Positive emotions	240	4.29	0.06	237	4.29	0.06	−0.01	0.909	−0.01	−0.13	0.15	0.387
Negative emotions	239	1.88	0.06	236	1.85	0.07	0.03	0.082	0.04	−0.13	0.17	0.449

M, adjusted mean; SE, standard error; significant effects are highlighted in bold.

The only significant average effect occurs at the 1-month follow-up: the average effect of the treatment on *negative emotions* at the 1-month follow-up is statistically significant (estimate −0.18; *p* = 0.020; Glass *d* = −0.24). Therefore, only H7 is confirmed and only for the 1-month follow-up. For the baseline x treatment interaction, two significant effects are found: for the *parent support and home life* scale at the post-measurement time point, the estimate for the average effect of treatment would change by −0.27 if the baseline value changed by one SD. The result indicates that children who report lower scores on the *parent support and home life* scale before the start of the program benefit more from the treatment at the post measurement time point. Equivalently, for the *self-esteem* scale, children with lower scores before the start of the treatment benefit more from the treatment at the 2-month follow-up.

### 3.2 Parent data results

The research hypotheses about the parents' assessment of their children were tested with 15 structural equation models for the five scales at three measurement time points. Table 7 shows the adjusted means and standard errors for the scales used per group and measurement time point, as well as the average effects of the treatment and the interaction between the baseline measurement and the treatment condition.

**Table 7:** Effects of the program – parents.

**Post**												
**Scale**	**Treatment**			**Control**			**Average effect**			**Baseline x Treatment**		
	***N***	***M***	***SE***	***N***	***M***	**SE**	**Estimate**	***p***	**Glass d**	**Estimate**	**SE**	***p***
Psychological wellbeing	50	51.8	1.23	36	49.5	1.87	2.33	0.301	0.25	−4.27	2.38	0.077
Moods and emotions	49	48.7	1.56	36	47.4	2.34	1.33	0.638	0.11	−2.91	2.94	0.324
Parent support and home life	36	48.5	1.34	50	49.4	1.92	−0.83	0.723	−0.12	−2.08	1.78	0.248
School environment	50	53.9	1.24	36	53.1	1.74	0.80	0.709	0.09	−1.93	2.19	0.381
Self-esteem	50	72.9	1.94	36	74.5	2.76	−1.60	0.637	−0.12	−3.73	4.00	0.354
**1-month follow-up**												
**Scale**	**Treatment**			**Control**			**Average effect**			**Baseline x Treatment**		
	***N***	***M***	***SE***	***N***	***M***	**SE**	**Estimate**	***p***	**Glass d**	**Estimate**	**SE**	***p***
Psychological wellbeing	51	53.5	1.17	51	49.4	1.27	**4.04**	**0.021**	**0.54**	0.85	1.85	0.645
Moods and emotions	51	47.9	1.52	51	48.5	1.64	−0.63	0.780	−0.05	−1.31	2.36	0.579
Parent support and home life	51	51.8	1.52	51	50.0	1.74	1.85	0.426	0.23	1.61	1.84	0.386
School environment	51	56.1	1.22	51	53.4	1.29	2.74	0.126	0.32	−0.98	1.99	0.622
Self-esteem	51	75.5	1.17	51	71.3	2.27	4.18	0.186	0.24	2.81	4.34	0.519
**2-month follow-up**												
**Scale**	**Treatment**			**Control**			**Average effect**			**Baseline x Treatment**		
	***N***	***M***	***SE***	***N***	***M***	***SE***	**Estimate**	***p***	**Glass d**	**Estimate**	**SE**	***p***
Psychological wellbeing	33	53.8	1.46	33	52.8	1.60	1.02	0.638	0.11	2.94	2.60	0.262
Moods and emotions	33	49.9	1.96	33	48.9	2.12	0.93	0.747	0.08	−0.87	3.36	0.797
Parent support and home life	33	50.8	1.69	33	50.4	1.92	0.36	0.889	0.05	0.07	1.81	0.971
School environment	32	53.1	1.70	33	55.9	1.79	−2.76	0.268	−0.30	0.05	2.65	0.986
Self-esteem	34	73.8	3.31	33	75.9	3.66	−2.07	0.677	−0.14	−1.12	6.50	0.854

M, adjusted mean; SE, standard error; significant effects are highlighted in bold.

The only significant average effect occurs at the 1-month follow-up: The average effect of the treatment on the parental assessment of their child's *psychological wellbeing* at the 1-month follow-up is statistically significant (estimate 4.04; *p* = 0.021; Glass *d* = 0.54). Therefore, only H1_parents_ is confirmed and only for the 1-month follow-up.

### 3.3 Subjective assessments of the program

The *children* of the treatment group were asked about their subjective assessments of the program at the post measurement point using seven *ad hoc* items. Table 8 shows the items in wording and the percentage response frequencies of the participating children (*n* = 208). In summary, the fourth graders perceived their participation in the happiness lessons as very positive (items 1, 2, 3, 4, and 5). Over 75% of the children reported learning something important for themselves during the happiness lessons (Item 6). Further engagement with the contents of the program in the families took place in about 48% according to the children (item 7).

**Table 8:** Subjective perception of the program – children.

**Item**		**True**		**Rather true**		**Probably true**		**Rather not true**		**Not true**	
1	I think it is a pity that the happiness lessons are over.	148	71%	29	14%	19	9%	5	2%	6	3%
2	I am glad that I participated in the happiness lessons.	145	70%	36	17%	18	9%	3	1%	6	3%
3	Overall, I enjoyed the happiness lessons.	142	69%	35	17%	19	9%	8	4%	3	1%
4	I felt good in the happiness lessons.	138	67%	40	19%	20	10%	7	3%	2	1%
5	If I could decide, there would be happiness lessons for all the children.	133	64%	29	14%	30	14%	10	5%	6	3%
6	I learned important things in the happiness lessons.	112	55%	46	23%	33	16%	7	3%	5	2%
7	In my family, we often talked about things from the happiness lessons.	57	27%	46	22%	38	18%	33	16%	34	16%

*N* = 208.

The *parents* of the treatment group were asked about their subjective perceptions of the program at the post measurement point using eight items. Table 9 shows the items in wording and the response frequencies of the participating parents (*n* = 60). In summary, parents perceived their child's participation in the happiness lessons as positive (items 1, 2, and 3). In particular, more than 80% of parents would make the content of the GlüGS project a permanent part of the school curriculum if they had a say in it (item 3). Over 65% of the parents believed that their child learned a lot of valuable things for himself or herself in the happiness lessons (item 4). The parent letters were considered valuable by over 65% (item 5). Satisfaction with the information about the project was over 75% (item 6). Less than half of the parents said that their families continued to engage with the content from the project (items 7 and 8).

**Table 9:** Subjective perception of the program – parents.

**Item**		**Completely true (1)**		**2**		**3**		**4**		**Not at all true (5)**	
1	I am glad that my child was able to participate in the GlüGS project.	39	65%	12	20%	8	13%	1	2%	-	
2	All in all, I am satisfied with the GlüGS project.	35	59%	18	31%	4	7%	2	3%	-	
3	If I could decide, I would make the content from the GlüGS project a permanent part of the school curriculum.	35	58%	15	25%	10	17%	-		-	
4	I believe that my child learned a lot of valuable things in the GlüGS project.	20	33%	18	30%	15	25%	4	7%	3	5%
5	The parent letters contained valuable information for me.	25	42%	15	25%	13	22%	5	8%	2	17%
6	I felt well informed about the project (cover letter, website, video, letters to parents).	35	58%	11	18%	9	15%	3	5%	2	3%
7	I applied some of the suggestions and happiness tips from the letters to parents.	9	15%	18	30%	15	25%	8	13%	10	17%
8	We often talked about the GlüGS project as a family.	8	13%	15	25%	13	22%	14	23%	10	5%

*N* = 60.

The class *teachers* were also asked about their perception of the program. From the 15 teachers we received 12 questionnaires back. Table 10 shows the items in wording and the response frequencies of the participating teachers. In summary, teachers perceived their participation in the happiness lessons as very positive (items 1, 2, 3, and 4). They felt well informed about the project (item 5), and learned valuable things for their own practice (item 6). About half of them continued to work with suggestions from the project to some extend (item 7), and a majority said that the project did not involve much extra work for them.

**Table 10:** Subjective perception of the program – teachers.

**Item**		**Completely true (1)**		**2**		**3**		**4**		**Not at all true (5)**	
1	I am glad that my class was able to participate in the GlüGS project.	9	75%	2	17%	1	8%	-		-	
2	All in all, I am satisfied with the GlüGS project.	6	50%	5	42%	1	8%	-		-	
3	If I could decide, I would make the content from the GlüGS project a permanent part of the school curriculum.	9	75%	3	25%	-		-		-	
4	I believe that the children of my class learned a lot of valuable things in the GlüGS project.	7	58%	5	42%	-		-		-	
5	I felt well informed about the project.	9	75%	2	17%	1	8%	-		-	
6	I learned valuable things from the GlüGS project for my own professional practice.	3	25%	8	67%	1	8%	-		-	
7	I have continued to work with suggestions from the project in my class.	1	9%	5	45%	4	36%	-		1	9%
8	Overall, taking part in the GlüGS project didn't involve much extra work for me.	4	33%	5	42%	2	17%	1	8%	-	

N = 12.

In the context of the very positive subjective perception of the program, it should be noted that all satisfaction items were formulated positively and could have provoked biases, for example, in relation to social desirability and demand characteristics.

### 3.4 Open questions

At post measurement point children, parents and teachers were also asked three open questions about their assessment of the project. The answers were summarized in terms of content and reported below with as little bias as possible, whereby individual single opinions were not taken into account in the presentation.

#### 3.4.1 Children's qualitative answers

A total of 172 (of a total of 242) children answered to the question “*What did you particularly like about the happiness lessons? Why?*” With 92 mentions, specific exercises from the program were mentioned frequently: the stories read aloud were mentioned most often, followed by the exercises Warm Shower Cards (giving compliments), Garden of Emotions (expressing emotions), Yellow Backpack (focusing on good things), Good Mood Alley (emotional contagion), and Dream Journey (relaxation), as well as the welcome and the closing ritual. A total of 23 responses were similar to “All, because it was fun.” A total of 35 children wrote about positive effects of the happiness lessons (e.g., “Now you also think about the little things,” “It makes you happier. Because you learn how to do it” or “I found it positive for life”). A total of 21 times it was said that good feelings arose during or after the happiness lessons. Eight children found it positive to do something different than “normal school”. The content and design of the lessons were mentioned seven times. Six children found the university students particularly great. Five children wrote that they appreciated talking about feelings at school. In response to the question “*Was there anything about the happiness lessons that you didn't like at all or that made you feel uncomfortable? Why?*” a total of 140 children responded in writing. The majority of these responses (102) were answers such as “no,” “never,” or “I liked everything”. specific exercises were mentioned a total of eight times (Happiness Diary, Dream Journey, and welcome and closing ritual). Two children did not like the homework and another two children stated that they did not feel good when expressing personal things. A total of 149 children responded to the question “*Is there anything else you would like to say to us?*” – however, 47 of them wrote something like “no” as an answer. In 52 responses, children expressed that they enjoyed the happiness lessons and had fun doing them (e.g., “I thought the happiness lessons were very very great, I felt good after the happiness lessons”). A total of 21 children expressed gratitude for the happiness lessons (e.g., “Thank you for taking the time for us, it was very very very much fun. Thank you so much!”). Eight children expressed that they thought it was a pity that the project was now over and 10 others called for the project to continue and expand (e.g., “Please make the happiness lessons into a happiness subject”). A total of 15 children wrote greetings or other expressions of sympathy to the university students (e.g., “I like you both very much”).

#### 3.4.2 Parent's qualitative answers

Equivalently to the children's questionnaire, the online questionnaire for the parents also contained three open questions. A total of 25 parents responded to the question “*Do you remember anything particularly positive about the GlüGS project? What?”* A total of 11 parents mentioned specific exercises, with the Warm Shower Cards (received compliments from classmates which could also be shown at home) being mentioned seven times. Four parents expressed appreciation for the general content or participation in the project, and another three mentioned the encouragement to be more aware of the good things in life as a particularly positive content. Two other parents found the letters to parents valuable (although two others stated here that they had not received any letters to parents). To the question “*Do you remember any difficulties associated with the GlüGS project?”* a total of 19 parents responded in writing, with seven of them answering “none” or similar. Five parents stated that their child had difficulties with a particular exercise in the project (3x Happiness Diary; 2x writing the Warm Shower Card for others) because of the amount of time required or because the demands of the exercise had been too high. The other comments referred to individual topics such as general problems of the child, too little time for the content at school or too little time for consolidation at home. The third question gave parents the opportunity to share *further comments, ideas, suggestions and other thoughts*. A total of 20 parents made use of this. Five parents explicitly thanked for the project and five others would like to see more content like this or a whole corresponding compulsory curriculum. Six of the comments included references to positive effects on themselves, the child or in the families. Two parents expressed their wish for better communication about the project. Overall, appreciation and sometimes even enthusiasm for the project was evident in many of the brief responses.

#### 3.4.3 Teachers' qualitative answers

To the four open questions at post measurements of the teachers' questionnaire 11 class teachers answered. In response to the question “*Do you remember anything particularly positive about the GlüGS project? What?*” four teachers mentioned the stories and two others the exercise Yellow Backpack. Three teachers reported that they were impressed by the insightful contributions of the children (e.g., “I was often touched by the meaningful answers/thoughts of many children”). Twice, the students' performance was emphasized positively. To the question “*Do you think the project will have a long-term impact? If yes, which ones? If not, why not?*” three responded positively and a further seven with the reservation that the content would need to be further deepened or repeated. In response to the question “*Is there anything you have taken out of the project? If so, what?*” seven teachers stated that they have taken away suggestions for their own lessons. Three explicitly stated that they wanted to focus more on the children's strengths in the future and three others have taken impulses for themselves. When asked about *suggestions for improvement or other comments*, three teachers replied that they would like to see more of this content in school. Four teachers made didactic comments and two others criticized the questionnaires used.

## 4 Discussion

In the present study, an existing program to increase the wellbeing of school children in fourth grade was condensed and adapted for use in elementary schools in the university context of teacher education. In a seminar course, university students were trained to lead in pairs 11 happiness lessons which were implemented in fourth grade classes in the presence of the teacher. Two renowned questionnaires on quality of life, for which norm data and versions for children and parents are available, were used for evaluation. In addition, a version of a questionnaire adapted for children was used to survey the frequency of positive and negative emotions. For subjective perceptions of the program, *ad hoc* items and open questions were asked.

It was postulated that the program would have a positive influence on the constructs *psychological wellbeing, moods and emotions, parent support and home life, school environment, self-esteem*, and the *frequency of positive and negative emotions*. These assumptions could be confirmed for the children only for one data point at the 1-month follow-up of the subscale *frequency of negative emotions*. For parents, a medium effect of the program on *psychological wellbeing* was found at 1-month follow-up. The two significant interaction effects in the children's data suggest that children with lower baseline levels in *parent support and home life* and *self-esteem* benefit more from the program. In contrast to these fewer than expected significant effects, the responses of participating children and parents on their subjective perception of the project indicate high satisfaction with the program and some heterogeneous improvements in children's behavior. Although it must be said, that these results may be positively biased due to social desirability and demand characteristics of the *ad hoc* items. In informal (and undocumented) exchanges with school administrators, teachers and parents, positive experiences with the pilot project and positive effects on, for example, class climate, social behavior or behavior of individual children were consistently mentioned. The quantitative results with the established instruments thus contradict the findings on positively evaluated Positive Education programs presented above, while the subjective evaluation of the program via qualitative responses and *ad hoc* items as well as other contextual information suggest undetected effects. Overall, the responses to the subjective perception of the program as well as the responses to the open questions draw a positive picture of the happiness lessons. The additional hypothesis of a positive perception of the program by the participating fourth graders can therefore be confirmed.

There are several possible explanations for the non-significant results of the present study. First, the intensity of the present intervention with 11 happiness lessons of 45 min each over a period of 3 months is substantially lower than, for example, the GNH curriculum evaluated by Adler (2016). There, over a period of 15 months, positive-psychological content was implemented not only in concrete Positive Education learning units but also in other subjects such as literature or biology at the participating schools. The content was imparted by trained teachers who took part in a ten-day training course beforehand. Against this background, the two effects found in this study can already be considered a good partial success. In her review, Waters (2011) named two common factors of successful positive psychology wellbeing programs: (1) infusion of positive psychology skills into the already established school subjects and (2) implementation of the intervention by the teachers. The first factor was also applied in the wellbeing curriculum from Adler (2016) mentioned above and it appears logical that more information, learning experiences, and application opportunities in more different contexts lead to stronger effects. Taking this factor further, it could lead to a whole school approach, which also includes other aspects such as the school's mission statement and the so-called hidden curriculum. Again, greater effects can be expected for interventions that also focus on the unofficial attitudes, values, and perspectives of all those involved in the school (cf. Waters, 2011). At the same time, however, such interventions also require significantly more resources to be realized – starting with convincing the staff of the need for an intensive and laborious change process. The program evaluated here offers the advantage of relieving teachers rather than causing them additional work and also requires only minimal financial resources. The involvement of the students counteracts the second common factor from Waters (2011) – but on the other hand, it reduces the workload for the teachers, who do not have to prepare or post-process the happiness lessons. Instead, they are able to observe their class in a new and motivating situation and gain new ideas for their own professional practice. Accordingly, the project could perhaps be a good starting point for a gradual school development process with a slower but experience-based warm-up phase for the stakeholders in the school. The engagement of newly trained students would also make it easier to continuously improve the program and thus also the impulses for the schools. Another side effect of our approach is that, at an early stage of their teacher training, students learn how valuable promoting wellbeing in schools can be and how to apply this in practice.

In their origins of happiness model, Clark et al. (2018) visualized that the wellbeing of children is influenced by many factors from which “schooling” is only one. Determinants are also found in the genes, but also other family variables such as parenting skills, parents (mental) health, income etc. Regardless of the average effectiveness of positive education programs, these variables can have an enormous impact on children's wellbeing. If these are too strongly negative, improving them will have a much greater impact on wellbeing than positive education programs of any intensity can have.

### 4.1 Limitations

In addition to these possible explanations for few significant effects found, another one could be based on the instruments used. In the analysis at item level (baseline), it is noticeable that a considerable proportion of the children selected the most positive response category in each case. The item with the lowest approval rate was “Have you been in a good mood” – here the highest answer category “always” was still chosen by 23.8%. The item with the highest approval rate was “Have you been happy about being alive” – here the large majority of 86.5% chose the highest category “very much.” Of the total 39 items, the highest category was selected by at least 60% of the children for 10 items and by <30% for only 5 items. Accordingly, the scope for possible improvement was limited by the high baseline values. On the other hand, as shown in Table 5, baseline data in this study is quite close to that of the norm sample for the kidscreen questionnaire and the instrument has already proven its' usefulness in detecting increasements in quality of life (Ravens-Sieberer et al., 2014).

Another limitation was the suboptimal survey situation in the classes, especially due to low human and financial resources of the project. The surveys were conducted at the first two measurement time points by the university students who also led the happiness lessons (both in the treatment and control classes) and mostly by other university students at the later measurement time points. Despite a short briefing on how to conduct the surveys, it could not be ensured that queries or problematic situations were handled similarly. Due to limited time slots, it was also not possible to ensure to the desired extent that all children completed the survey properly, which led to forgotten back pages or slips in the line and thus missing values in the analysis. In addition, in several classes there were children who had recently fled from the Ukraine, some of whose German language skills were very limited. Some received help in translations by fellow children, others were told that they did not need to participate. These limitations applied equally to the treatment and control classes.

As a last point, diffusion of treatment is another possible explanation for the limited gains of the intervention. Since in most cases each school participated with two fourth-grade classes, one of which was assigned to the treatment group and the other to the waiting control group, it is very likely that the fourth graders, their parents and their class teachers of the treatment group shared information and experiences with those of the control group.

### 4.2 Practical implications

Despite the low number of significant effects, the experiences and feedback on the project are very positive, as can be seen in the qualitative statements and the satisfaction items. Several measures can be derived as practical implications for further development of the program. As recommended by Waters (2011), greater effects of Positive Education programs can be expected if the whole school community with all stakeholders is involved. The parent letters have already been positively highlighted in some of the feedback. The information they contain could probably be distributed even more effectively through e-mail or messenger distribution lists. The effect could be even greater if content could be shared in the form of very short videos (e.g., as YouTube Shorts). Information sessions or short trainings for parents would also be desirable, although great care must be taken here to create an attractive offering – and not another undesirable compulsory time for school affairs. Following the whole school approach, it would also be beneficial to train the teachers and other pedagogical staff at the school. The first goal should be to increase the wellbeing of the participants (for an example of an evaluated training, see Rahm and Heise, 2019) and only in the next step to address didactic methods and organizational possibilities of how to convey wellbeing-enhancing content to the children. In the best case, a school-wide and structured organizational development process could be implemented in which the school culture, mission statement, leadership style, etc. are also aligned to promote the wellbeing of all school members. A well-known best practice example of such a development process is the Geelong Grammar School in Australia where positive education content is taught 2 h every week in every year (Norrish, 2015). From the interaction effects found in this study, it can also be assumed that the present program could achieve more effect for children with lower baseline values. In further development of the program, possibilities for internal differentiation should therefore also be considered. Reflection tasks, for example, could be offered with different levels of difficulty.

### 4.3 Further research

The dynamic research field of Positive Education continues to face major challenges - in particular, there has been a lack of a sound theoretical framework with consistent conceptualizations of key constructs such as wellbeing and flourishing (Cabanas and González-Lamas, 2022). In the present study, a program developed in educational practice was evaluated using existing measurement instruments. It seems more promising if the target constructs of treatment and measurement instrument are built on the same theoretical foundation. To gain insight into the success (or failure) of Positive Education programs in the current setting, qualitative research approaches might be especially valuable. In the present study, quantitative findings could already be usefully supplemented and put into perspective by content-analytical evaluation of some open questions. For a more holistic view of the potentials and effects of Positive Education programs, content analysis of structured research interviews with participating children, parents, and teachers seems particularly promising.

### 4.4 Final remarks

Although the present Positive Education program was relatively short with 11 school hours, two statistically significant effects were found at the 1-month follow-up. Qualitative comments and responses to satisfaction items from children, parents, and teachers speak for a success of the project and encourage expanding this and similar programs in a variety of ways and further studying them for effectiveness. In particular, the implementation of more learning units over a longer period of time and a stronger anchoring in the entire school culture with the involvement of parents seem promising for the sustainable promotion of greater wellbeing for everyone at school.

## Data availability statement

The raw data supporting the conclusions of this article will be made available by the authors, without undue reservation.

## Ethics statement

The studies involving humans were approved by the Ethics Committee of the Faculty of Life Sciences, Technische Universität Braunschweig (FV-2022–19). The studies were conducted in accordance with the local legislation and institutional requirements. Written informed consent for participation in this study was provided by the participants' legal guardians/next of kin.

## Author contributions

TR: Conceptualization, Funding acquisition, Investigation, Methodology, Project administration, Supervision, Visualization, Writing – original draft, Writing – review & editing. NO: Data curation, Formal analysis, Methodology, Resources, Writing – original draft, Writing – review & editing. AM: Formal analysis, Methodology, Software, Supervision, Validation, Writing – review & editing.

## Footnotes

^1^When using the word parents in this article, all legal guardians are included. In materials used in the project, both groups were addressed equally.
